# Impact of Time Since Diagnosis and Age on Fracture Risk in Young Adults With Type 1 and Type 2 Diabetes

**DOI:** 10.1002/kjm2.70112

**Published:** 2025-09-27

**Authors:** Tien‐Ching Lee, Sung‐Yen Lin, Chung‐Hwan Chen, Pei‐Shan Ho

**Affiliations:** ^1^ Department of Orthopedics, College of Medicine Kaohsiung Medical University Kaohsiung Taiwan; ^2^ Department of Orthopedics Kaohsiung Medical University, Hospital, Kaohsiung Medical University Kaohsiung Taiwan; ^3^ Regenerative Medicine and Cell Therapy Research Center Kaohsiung Medical University Kaohsiung Taiwan; ^4^ Orthopaedic Research Center, College of Medicine Kaohsiung Medical University, Hospital, Kaohsiung Medical University Kaohsiung Taiwan; ^5^ Department of Orthopedics Kaohsiung Medical University Gangshan Hospital Kaohsiung Taiwan; ^6^ Department of Orthopedics, School of Post‐Baccalaureate Medicine, College of Medicine Kaohsiung Medical University Kaohsiung Taiwan; ^7^ Faculty of Dental Hygiene College of Dental Medicine, Kaohsiung Medical University Kaohsiung Taiwan

To the Editor,

Diabetes mellitus (DM) is associated with increased fracture risk through alterations in bone mass, quality, and microarchitecture [1, 2, 3]. While fracture risk in older adults with DM is well established, data are limited for younger adults, who may have many decades of risk ahead [3, 4, 5]. Furthermore, the influence of diabetes type—type 1 (T1DM) versus type 2 diabetes (T2DM)—on fracture timing in these younger populations remains unclear.

We examined this question using Taiwan's nationwide Longitudinal Generation Tracking Database 2000 (LGTD 2000). This database, derived from the National Health Insurance program covering over 99% of the population, contains comprehensive longitudinal medical claims data. We identified individuals aged 20–55 years with newly diagnosed T1DM or T2DM between 2000 and 2019. DM diagnoses were confirmed by diagnostic coding and at least one antidiabetic prescription. Patients with prior fractures, fracture codes linked to traffic accidents, or incomplete demographic data were excluded.

Because T1DM is relatively uncommon in this age range, we matched each T1DM patient with four T2DM patients by age and sex to improve statistical efficiency. Fracture incidence was identified from hospital admissions or outpatient visits with fracture codes. We compared proportions using chi‐squared tests, means using *t*‐tests, and cumulative fracture‐free survival using Kaplan–Meier analysis with log‐rank testing. Cox proportional hazards regression was used to estimate the association between diabetes type, Charlson Comorbidity Index, and fracture risk. The study adhered to the Helsinki Declaration and received Institutional Review Board approval (KMUHIRB‐E(II)‐20210238).

The final analysis included 497 young adults with T1DM and 1977 with T2DM. Mean follow‐up was 14.0 years for the T1DM group and 10.5 years for the T2DM group. Fractures occurred in 3.1% of T1DM patients compared with 1.2% of T2DM patients. In unstratified Kaplan–Meier analysis, time to fracture was not significantly different between groups. However, after stratifying by age, fractures occurred significantly earlier in individuals with T1DM (Figure 1). Cox regression models showed a trend toward higher fracture hazard in T1DM, although statistical significance was not reached, likely reflecting the modest number of fracture events.

**FIGURE 1:**
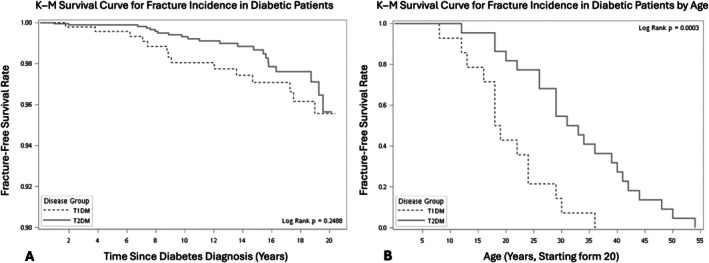
(A) Kaplan–Meier survival curve for fracture incidence in diabetic patients; (B) Kaplan–Meier survival curve for fracture incidence in diabetic patients by age.

Longer cumulative exposure to hyperglycaemia, diabetes‐related microvascular complications, impaired bone quality, and possible differences in physical activity and nutritional status may contribute to earlier skeletal fragility in T1DM. Limitations of this study include the lack of body mass index, bone mineral density, lifestyle factors, and medication details, as well as no non‐diabetic control group. The relatively small T1DM sample size also reduced power for subgroup analyses, including by fracture type.

Despite these limitations, our findings suggest that young adults with T1DM may sustain fractures earlier than those with T2DM. Clinicians should consider initiating bone health evaluation and preventive measures earlier in this population. Prospective studies with more comprehensive data are needed to confirm these findings and clarify underlying mechanisms.

## Conflicts of Interest

The authors declare no conflicts of interest.

## Supporting information


**Figure S1:** Flowchart of study population selection (T1DM, type 1 diabetes mellitus; T2DM, type 2 diabetes mellitus).

**Table S1:** Baseline characteristics of the matched cohort of type 1 diabetes mellitus (T1DM) and type 2 diabetes mellitus (T2DM) patients.CCI, Charlson Comorbidity Index.

**Table S2:** Characteristics of young adults (20–55 Years) with type 1 diabetes mellitus (T1DM) and type 2 diabetes mellitus (T2DM) with fracture occurrence.

**Table S3:** Cox proportional hazards regression analysis of Fracture risk in young adults with diabetes. CCI, Charlson Comorbidity Index.

**Table S4:** Demographic characteristics of the study participants with type 1 diabetes mellitus (T1DM) and type 2 diabetes mellitus (T2DM). (SD, standard deviation; CCI, Charlson Comorbidity Index).

## Data Availability

The data that support the findings of this study are available on request from the corresponding author. The data are not publicly available due to privacy or ethical restrictions.
