# Supplementary material for: Impact of Time Since Diagnosis and Age on Fracture Risk in Young Adults With Type 1 and Type 2 Diabetes
Source: Kaohsiung J Med Sci. 2025 Sep 27;42(3):e70112. doi: 10.1002/kjm2.70112 (PMC12955857; doi:10.1002/kjm2.70112)
Supplement: Supplementary file 1 — Figure S1: Flowchart of study population selection (T1DM, type 1 diabetes mellitus; T2DM, type 2 diabetes mellitus). [file KJM2-42-e70112-s001.docx]

Supplementary Figure 1. Flowchart of Study Population Selection

Patient diagnosed with T1DM in 2000-2019 (n=19,459)

Excluding:

1. Patients without prescription of insulin for 3 or more times (n=15,679)

2.Ppatients without the certificate of major illness (n=2,709)

3. Missing demographic data(n=2)

T1DM Patients (2000-2019) with ≥3 Insulin Prescriptions/Year and Catastrophic Illness Registry Confirmation (n=1,069)

Excluding patients with fracture before T1DM diagnosed (n=81)

T1DM group (n=988)

1:4 Age/Gender Matching (20-55 Years at DM Diagnosis)

T1DM (Cases): n=497

T2DM (Controls): n=1977

Patient diagnosed with T2DM in 2000-2019 (n=392,074)

Excluding:

1. Patients diagnosed with T1DM (n=17,120)

2. Patients without prescription of antidiabetic drugs (n=216,033)

3. Missing demographic data(n=2,281)

T2DM Patients (2000-2019) with ≥1 Antidiabetic Prescription/Year (n=156,640)

Excluding patients with fracture before T2DM diagnosed (n=10,091)

T2DM group (n=146,549)
