# Supplementary material for: Impact of Time Since Diagnosis and Age on Fracture Risk in Young Adults With Type 1 and Type 2 Diabetes
Source: Kaohsiung J Med Sci. 2025 Sep 27;42(3):e70112. doi: 10.1002/kjm2.70112 (PMC12955857; doi:10.1002/kjm2.70112)
Supplement: Supplementary file 2 — Table S1: Baseline characteristics of the matched cohort of type 1 diabetes mellitus (T1DM) and type 2 diabetes mellitus (T2DM) patients.CCI, Charlson Comorbidity Index. [file KJM2-42-e70112-s003.docx]

| Variable | Group | N | Mean ± SD | P-Value |
| --- | --- | --- | --- | --- |
| Fracture Occurrence | |  |  | <0.001 |
|  | T1DM | 14 (3.10%) |  |  |
|  | T2DM | 22 (1.20%) |  |  |
| DM Diagnosis Age (years) | | 36 |  | 0.002 |
|  | T1DM |  | 30.21 ± 9.03 |  |
|  | T2DM |  | 41.05 ± 9.43 |  |
| Fracture Diagnosis Age (years) | | 36 |  | 0.001 |
|  | T1DM |  | 40.50 ± 7.62 |  |
|  | T2DM |  | 52.77 ± 11.22 |  |
| Disease Duration (years) | | 36 |  | 0.502 |
|  | T1DM |  | 10.50 ± 5.30 |  |
|  | T2DM |  | 11.72 ± 5.24 |  |

Table 1. Characteristics of Young Adults (20-55 Years) with T1DM and T2DM with Fracture Occurrence
