# Supplementary material for: Impact of Time Since Diagnosis and Age on Fracture Risk in Young Adults With Type 1 and Type 2 Diabetes
Source: Kaohsiung J Med Sci. 2025 Sep 27;42(3):e70112. doi: 10.1002/kjm2.70112 (PMC12955857; doi:10.1002/kjm2.70112)
Supplement: Supplementary file 3 — Table S2: Characteristics of young adults (20–55 Years) with type 1 diabetes mellitus (T1DM) and type 2 diabetes mellitus (T2DM) with fracture occurrence. [file KJM2-42-e70112-s002.docx]

|  |  |  | T1DM^1^ | | T2DM | | P-Value |
| --- | --- | --- | --- | --- | --- | --- | --- |
|  |  | Total | Number | % | Number | % |  |
|  |  | 147537 | 988 | 0.7 | 146549 | 99.3 |  |
| Gender | Female | 67248 | 569 | 57.6 | 66679 | 45.5 | <0.0001 |
|  | Male | 80289 | 419 | 42.4 | 79870 | 54.5 |  |
| Age of DM diagnosis | Mean (±SD) | 147537 | 22.49±13.67 | | 56.20±12.77 | | <0.0001 |
| CCI | 0 | 102028 | 852 | 86.2 | 101176 | 69 | <0.0001 |
|  | 1 | 28393 | 102 | 10.3 | 28291 | 19.3 |  |
|  | 2+ | 17116 | 34 | 3.4 | 17082 | 11.7 |  |
| Any fracture | No | 137315 | 969 | 98.1 | 136346 | 93 | <0.0001 |
|  | Yes | 10222 | 19 | 1.9 | 10203 | 7 |  |
| Age of fracture | Mean (±SD) | 10222 | 37.53±15.25 | | 73.59±10.82 | | <0.0001 |
| Follow-up duration | Mean (±SD) | 147537 | 13.33±5.98 | | 11.02±5.94 | | <0.0001 |

Supplementary table 1. Demographic characteristics of the study participants with Type 1 Diabetes Mellitus (T1DM) and Type 2 Diabetes Mellitus (T2DM). (T1DM: Type 1 Diabetes Mellitus; T2DM: Type 2 Diabetes Mellitus; SD: Standard Deviation; CCI: Charlson Comorbidity Index)
