# Supplementary material for: Impact of Time Since Diagnosis and Age on Fracture Risk in Young Adults With Type 1 and Type 2 Diabetes
Source: Kaohsiung J Med Sci. 2025 Sep 27;42(3):e70112. doi: 10.1002/kjm2.70112 (PMC12955857; doi:10.1002/kjm2.70112)
Supplement: Supplementary file 4 — Table S3: Cox proportional hazards regression analysis of Fracture risk in young adults with diabetes. CCI, Charlson Comorbidity Index. [file KJM2-42-e70112-s005.docx]

| Variable | Group | N | % | P-Value |
| --- | --- | --- | --- | --- |
| Total number |  | 2474 | 100 |  |
|  | T1DM | 497 | 20.1 |  |
|  | T2DM | 1977 | 79.9 |  |
| Gender |  |  |  | 0.94 |
|  | Female | 1415 | 57.2 |  |
|  | Male | 1059 | 42.8 |  |
| DM Diagnosis Age (years) | | 2474 |  | 0.879 |
|  | T1DM |  | 32.55 ± 9.13 |  |
|  | T2DM |  | 32.62 ± 9.10 |  |
| Follow-up (years) | | 2474 |  | <0.001 |
|  | T1DM |  | 13.96 ± 6.11 |  |
|  | T2DM |  | 10.51 ± 5.81 |  |
| CCI Index |  |  |  | 0.1771 |
|  | 0 | 1998 | 80.8 |  |
|  | 1 | 369 | 14.9 |  |
|  | 2+ | 107 | 4.3 |  |
| Any Fracture | |  |  | 0.0037 |
|  | No | 2271 | 91.8 |  |
|  | Yes | 203 | 8.2 |  |

Supplementary table 2. Baseline Characteristics of the Matched Cohort of T1DM and T2DM Patients. (CCI: Charlson Comorbidity Index)
