# Supplementary material for: Impact of Time Since Diagnosis and Age on Fracture Risk in Young Adults With Type 1 and Type 2 Diabetes
Source: Kaohsiung J Med Sci. 2025 Sep 27;42(3):e70112. doi: 10.1002/kjm2.70112 (PMC12955857; doi:10.1002/kjm2.70112)
Supplement: Supplementary file 5 — Table S4: Demographic characteristics of the study participants with type 1 diabetes mellitus (T1DM) and type 2 diabetes mellitus (T2DM). (SD, standard deviation; CCI, Charlson Comorbidity Index). [file KJM2-42-e70112-s004.docx]

| Variable | Category | Adjusted Hazard Ratio (AHR) | 95% Confidence Interval (CI) | P-value |
| --- | --- | --- | --- | --- |
| DM Type | Type 2 DM | 1 (Reference) | - | - |
|  | Type 1 DM | 2.12 | 0.91 - 4.95 | 0.0816 |
| CCI Index | 0 | 1 (Reference) | - | - |
|  | 1+ | 1.15 | 0.32 - 4.12 | 0.8302 |

Table 3. Cox Proportional Hazards Regression Analysis of Fracture Risk in Young Adults with Diabetes. CCI: Charlson Comorbidity Index.
